# Low-Field NMR Study of Shortcake Biscuits with Cricket Powder, and Their Nutritional and Physical Characteristics

**DOI:** 10.3390/molecules26175417

**Published:** 2021-09-06

**Authors:** Krzysztof Smarzyński, Paulina Sarbak, Przemysław Łukasz Kowalczewski, Maria Barbara Różańska, Iga Rybicka, Katarzyna Polanowska, Monika Fedko, Dominik Kmiecik, Łukasz Masewicz, Marcin Nowicki, Jacek Lewandowicz, Paweł Jeżowski, Miroslava Kačániová, Mariusz Ślachciński, Tomasz Piechota, Hanna Maria Baranowska

**Affiliations:** 1 Students’ Scientific Club of Food Technologists, Poznań University of Life Sciences, 31 Wojska Polskiego St., 60-624 Poznań, Poland; krzysztof.smarzynski@gmail.com (K.S.); paulina.sarbak@onet.pl (P.S.); 2 Department of Food Technology of Plant Origin, Poznań University of Life Sciences, 31 Wojska Polskiego St., 60-624 Poznań, Poland; przemyslaw.kowalczewski@up.poznan.pl (P.Ł.K.); maria.rozanska@up.poznan.pl (M.B.R.); katarzyna.polanowska@up.poznan.pl (K.P.); dominik.kmiecik@up.poznan.pl (D.K.); 3 Department of Technology and Instrumental Analysis, Poznań University of Economics and Business, Al. Niepodległości 10, 61-875 Poznań, Poland; iga.rybicka@ue.poznan.pl; 4 Department of Gastronomy Science and Functional Food, Poznań University of Life Sciences, 31 Wojska Polskiego St., 60-634 Poznań, Poland; monika.fedko@up.poznan.pl; 5 Department of Physics and Biophysics, Poznań University of Life Sciences, 38/42 Wojska Polskiego St., 60-637 Poznań, Poland; lukasz.masewicz@up.poznan.pl; 6 Institute of Agriculture, University of Tennessee, 370 Plant Biotechnology Building, 2505 EJ Chapman Drive, Knoxville, TN 37996-4560, USA; mnowicki@utk.edu; 7 Department of Production Management and Logistics, Poznan University of Technology, 2 Jacka Rychlewskiego St., 60-965 Poznań, Poland; jacek.lewandowicz@put.poznan.pl; 8 Institute of Chemistry and Technical Electrochemistry, Poznan University of Technology, Berdychowo 4, 60-965 Poznań, Poland; pawel.jezowski@put.poznan.pl (P.J.); mariusz.slachcinski@put.poznan.pl (M.Ś.); 9 Department of Fruit Sciences, Viticulture and Enology, Faculty of Horticulture and Landscape Engineering, Slovak University of Agriculture in Nitra, Tr. A. Hlinku 2, 949 76 Nitra, Slovakia; miroslava.kacaniova@gmail.com; 10 Department of Bioenergy and Food Technology, Institute of Food Technology and Nutrition, University of Rzeszow, Cwiklinskiej 1, 35-601 Rzeszow, Poland; 11 Department of Agronomy, Poznań University of Life Sciences, 11 Dojazd St., 60-631 Poznań, Poland; tomasz.piechota@up.poznan.pl

**Keywords:** *Acheta domesticus*, amino acids composition, cookies with insects, edible insects, fatty acids, nutritional value, minerals, ^1^H NMR, water dynamics

## Abstract

The growing human population renders challenges for the future supply of food products with high nutritional value. Here, we enhanced the functional and nutritional value of biscuits, a popular sweet snack, by replacing the wheat flour with 2%, 6%, or 10% (*w*/*w*) cricket powder. Consumer acceptance ratings for reference and 2% augmented cookies were comparable, whereas the higher levels of enhancement received inferior consumer scores. This relatively small change in biscuit recipe provided significant and nutritionally desirable enhancements in the biscuits, observed in a series of analyses. An increase in the protein content was observed, including essential amino acids, as well as minerals and fat. This conversion also affected the physical properties of the biscuits, including hardness, and water molecular dynamics measured by ^1^H NMR. Cricket powder-augmented biscuits join the line of enhanced, functionally superior food products. This and similar food augmentation provide a viable scenario to meet the human food demands in the future.

## 1. Introduction

The growing awareness of consumers regarding proper nutrition makes them look for food that is right for them. A balanced diet ensures that the demand for macronutrients is met in the right proportions. It is important that the consumed food meets the energy and physiological needs in the right portions, but at the same time ensuring a sufficient amount of micronutrients necessary for the proper functioning of our body [1,2,3,4]. Numerous recent studies have focused on the enrichment of food products in various bioactive compounds [5]. Improving the nutritional value was also attempted by decreasing the fat and sugar content or increasing the protein content [6,7,8]. The changing demands of consumers looking for a “healthier” snack led to attempts to improve its nutritional value and functional properties. Even though biscuits are not regarded as a healthy choice, they are eagerly consumed around the world. The market of biscuits is constantly growing, which was particularly observed in last months of COVID-19, e.g., in the United States [9,10]. Additionally, in the United Kingdom, the average consumption of confectionery products remains at the high level of 123–137 g per person per week in 2008–2019 [11]. Therefore, biscuits, being one of the world’s most popular staple sweets, are considered a convenient food matrix for modification of their recipe by incorporation of various ingredients. Improvements in their nutritional value is achieved by adding whole grains or raw materials rich in dietary fiber, as well as by increasing the content of protein or minerals [12,13,14].

The FAO-estimated population growth to 9 billion in 2050 poses new challenges for food producers [15]. One of the main challenges will be to provide not only the right amount of food, but also an adequate supply of protein. The application of an unconventional source of protein—cricket powder (CP)—seems a promising approach to food for fortification with protein, vitamins, minerals such as Ca, Mg, K, Fe, Cu, Mn, and Zn, and dietary fiber [16,17,18]. The replacement of wheat flour with CP affects changes in the quality and digestibility of the product’s protein as well as the desirable essential amino acids profile [19]. Our previous investigations were conducted on enhancing the nutritional value of various food products: muffins, gluten-free bread, pasta, and pork pâtés by their supplementation with CP [20,21,22,23]. Interestingly, texture analysis showed that in the case of gluten-free bread, the replacement of starch by CP in the amount of up to 6% resulted in a reduction in firmness, likely due to the emulsifying properties of cricket proteins [22]. A similar observation was also found for muffins [20]. Moreover, the addition of CP reduced cooking losses and caused a significant increase in the firmness of cooked pasta samples, underscoring the high quality of the CP-enriched pasta [24,25].

LF NMR is a method designed to study the dynamics of protons, that can be employed in numerous applications [26]. Recently, there has been increasing interest in the application of LF NMR for food analysis [27,28]. The main reason for that is the possibility to study different processes in model food systems, including gelatinization [29,30], retrogradation [31] or hydratation of starch [32], lipid oxidation [33], and enzymatic modification of proteins [34]. Moreover, it is useful in the analysis of complex food matrices, as proton fractions of water, lipids, or polysaccharides tend to form separate populations that relax at significantly different rates. This allows for observation of interactions that may occur as the product ages or is reformulated. Therefore, LF NMR has proved to be an useful tool in the quality design of emulsions [35,36], bread [37], dough [38], pâté [21,39], and many other food products [40]. Considering the advantages of CP, the importance of enriching food products, and the many changes induced by enrichment and the usefulness of the LF NMR technique in the analysis of food, this investigation was carried out to evaluate how an addition of various levels of CP influenced the nutritional value, consumer acceptance, textural properties, and water behavior on a molecular level of shortcake biscuits.

## 2. Results and Discussion

### 2.1. Consumer Study

**Figure 1:**
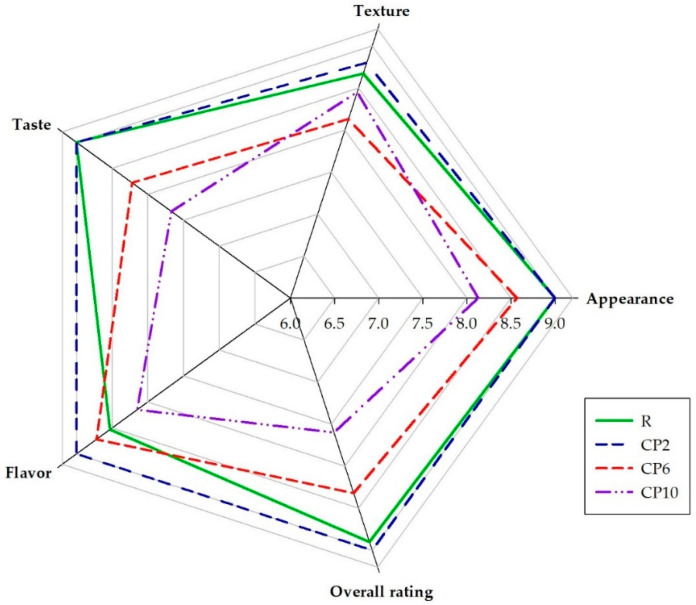
Results of consumer acceptance study. R—reference biscuits; CP2, CP6, and CP10—biscuits with 2%, 6%, and 10% of wheat flour replacement with CP, respectively.

The use of insects to enrich food may be negatively perceived by consumers [41]. It is extremely important to raise consumer awareness and identify potential health benefits [42,43,44]. For this reason, this study aimed to assess what level of replacement of wheat flour (WF) with CP in biscuits would be acceptable (Figure 1). There were no changes in the ratings of taste, texture, appearance, or the overall desirability of CP2 biscuits compared to reference biscuits (R); however, further increases in the replacement of WF with CP resulted in a significant reduction in the consumer acceptance scores awarded. In the case of the flavor evaluation, for both CP2 and CP6, the scores were significantly higher than for R. A small addition of CP significantly improved the flavor of the cookies. Commercial CP was used in this study, but in order to obtain CP, crickets were processed sequentially before being ground, including steaming, roasting, frying, and drying [45]. Technological treatment of insects can significantly improve the aroma of the resultant CP [46], and thus increase the consumer acceptance of such enhanced products. However, a 10% replacement of WF with CP resulted in an unpleasant, odd smell, which consumers indicated as undesirable. As reported by Grossmann et al. [47], most of the volatile odor-active compounds of crickets have been described as green, earthy or potato-mushroom, but have also been associated with a description of the smell of fat, sweat, cheese or popcorn. The volatile phenols present in crickets are responsible for the smell of smoke and feces. Therefore, too high a concentration of compounds present in crickets is unacceptable. With the increase in the conversion of WF to CP, biscuits more and more resembled wholemeal flour biscuits (see Section 2.2) and although such products are commonly considered to be more healthy [48], unfortunately this did not meet with growing marks in the consumer assessment of the appearance of biscuits. The evaluation of the texture of the biscuits has also changed. As in the case of flavor and appearance, texture scores also decreased with increasing WF to CP conversion (above 6%). Gluten proteins present in WF are responsible for creating the appropriate structure of cereal products [49]. Reducing its share in biscuits with the addition of CP resulted in an increase in their crispness and brittleness compared to biscuits without CP. Replacing WF with CP in the amount of 2% did not cause any significant changes in the taste assessment. Burt et al. suggest that the primary problem with the use of crickets in food production in Western cultures is a psychological one [50]; thus, on the basis of the obtained results, the 2% addition of CP could be fully acceptable, and the obtained shortcake biscuits could be successfully introduced to the market.

### 2.2. Biscuits Appearance

**Figure 2:**
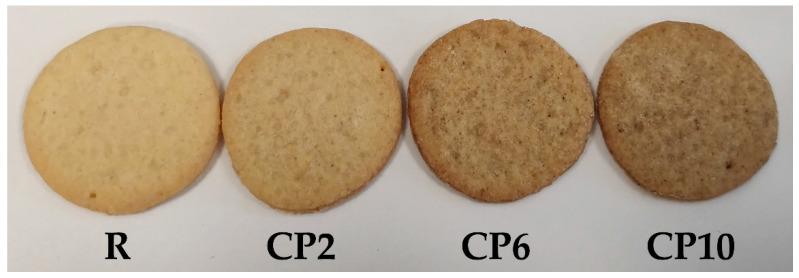
Biscuits with CP: R—reference biscuits; CP2, CP6, and CP10—biscuits with 2%, 6%, and 10% of wheat flour replacement with CP, respectively.

The use of CP in the recipe of shortcake biscuits caused changes in the consumer assessment, including when assessing for their appearance. This may be due to the discoloration of the final products, readily visible to the naked eye (Figure 2).

The color component analysis showed a significant darkening of the biscuits with CP (Table 1). The more WF was replaced with CP, the darker the biscuits became (lowering the L* value). The increase in the proportion of protein and the high-temperature process of baking the biscuits caused the formation of colored melanoid-forming products, among others in a Maillard reaction, but biscuits also can become darker due to the carbohydrate transformation including caramelization [51,52]. It was observed that the color of biscuits with the addition of CP gradually shifted in the red and blue directions (increase in red saturation (a*) and a decrease in yellow saturation (b*)). This phenomenon contributed to the initial increase in the whiteness index of the biscuits (CP2), that was neglected at higher CP content due to a more substantial lightness decrease. Similar changes were observed by Zielińska and Pankiewicz [53] in cookies enriched with *Tenebrio molitor*, as well as in other cereal products enriched with CP [20,24,54].

Total color difference analysis (ΔE) confirmed that the color changes caused by the addition of CP are significant. According to Mokrzycki and Tatol [55], the higher the ΔE value, the easier it is to observe the color difference, and untrained people can spot slight differences above ΔE = 2.0 and clear differences above ΔE = 3.5. This was reflected by the reduced appearance scores in the consumer analysis of biscuits containing elevated amounts of CP (6 and 10%) and similar ones for and CP2 (Figure 1).

### 2.3. Nutritional Value

Insects are widely described as a good source of protein, fat and minerals [56,57,58], so the use of CP can improve the nutritional value of a biscuit recipe. An increase in protein, fat, and ash content was observed, along with an increase in the conversion of WF to CP (Table 2). The consequence of the observed increases in their content was a gradual reduction in carbohydrate content. Additionally, an increase in the energy value of the cookies was observed. The most desirable biscuit, CP2, had more energy, fat, and protein than an average commercial biscuit. The 50 g portion of CP2 biscuits (about seven pieces) had realized 12% of the reference intake for energy and fat and, 8% for protein, and 14% for carbohydrates, being a nutritionally attractive sweet snack [59]. The moisture content did not differ statistically significantly.

The content of minerals: Ca, Mg, K, Na, Cu, Fe, Mn, and Zn is presented in Table 3. As CP is an important source of minerals, its addition to biscuits increased their elemental profile (except for Na) [19]. The content of most minerals was higher in biscuits with CP addition than in a commercial sample. The most significant differences between CP2 and a control sample were noticed for Ca (23%), Fe (12%), Mn (14%), and Zn (16%). For Mg and K the content changed by 6% and 7%, respectively. However, a nutritional claim on “source of mineral” could only apply to Mn in CP6 or Cu, Mn, and Zn in CP10 which were scored significantly lower in a sensory test [59,60]. The content of Na was comparable (302–323 mg/100 g) in all biscuits and resulted from the salt (sodium chloride) addition to the biscuit dough. Generally, the worldwide intake of Na is above nutritional recommendations, so it is suggested to lower its content in food products [61]. On the other hand, salt plays an extremely important role in sensory attributes of food products, so it is added to most of food categories. All products under the study, despite delivering 10–11% of adequate intake (AI) for Na in 50 g portion, would fulfill the clearly defined and rigorous latest British targets for salt reduction (maximum of 340 mg of Na/100 g in a category of biscuits) [62]. Moreover, the portion of CP2 biscuits provided 10% of nutrient reference value/adequate intake (NRV/AI) for Mn, 4% for Zn, 2% for Ca, Cu, Fe, and K, and 1% for Mg. In general, bakery confectionary products are not regarded as a source of minerals, so those developed with CP addition can be regarded as a healthier option than commercial ones.

The literature data indicate that crickets are a good source of fat; therefore, a change in the fatty acid profile in finished products was expected. Udomsil et al. [63] indicated that in the fats of crickets, the most abundant are saturated fatty acids (SFA), mainly palmitic acid (C16:0) and stearic acid (C18:0), and of monounsaturated fatty acids (MUFA), oleic acid (C18:1). There are also polyunsaturated fatty acids (PUFA), such as linolenic acid (C18:3) and linoleic acid (C18:2). Importantly, other studies have shown that the fatty acid profile does not differ across the tissues of the cricket that are eaten [64]. The test results confirmed the expected changes in the fatty acid profile (Table 4). A slight increase in the share of MUFA and PUFA was observed along with the increase in the replacement of WF with CP. Unfortunately, due to the use of large amounts of baking margarine in the recipe of cookies (see Section 3.1) produced from vegetable oils in varying proportions (palm, rapeseed, sunflower), it cannot be concluded that the nutritional value of CP cookies in the context of fatty acids is improved. Nevertheless, it can be expected that, similar to other low-fat products (e.g., pasta or bread), it will be possible to improve the nutritional value of the biscuits.

The amino acid profile is presented in Table 5. Along with the increase in the amount of CP in the biscuit recipe, a higher content of all analyzed amino acids was observed, except for phenylalanine and methionine. It is well known that the major amino acids in cereal prolamins are proline and glutamine [65], which is in line with the results of our research. The lowest-content essential amino acid in grains, in particular wheat, is lysine, and next up is threonine [66]. It has been noticed that even a 2% incorporation of CP into biscuit formula led to an increase in the content of essential amino acids by 13.6%. In comparison to the control sample (R), the content of lysine in the samples CP2, CP6, CP10 increased by almost 40%, 83.5%, and 108.3%, respectively. Moreover, in the case of analyzed biscuits samples, the concentration of threonine increased by an average of 31.3%. It should be noted that the higher lysine and arginine contents led to increased susceptibility of flour to the progress of the Maillard reaction [67]. The drawback is that some of the Maillard reaction products (MRPs) are currently suspected to have deleterious health effects. The accumulation of MRPs in vivo has been implicated as a major pathogenic process in diabetic complications and other disorders, such as atherosclerosis, Alzheimer’s disease, and normal aging [68].Thus, due to the possibility of the potentially harmful Maillard reaction compounds formation, it is worth noting to control their levels by the recipe’s modification, e.g., adding functional ingredients and/or different flours sources, especially in cereal products such as cereal products biscuits, and bread [69].

### 2.4. Physical Properties

The physical properties of the obtained biscuits were analyzed by characterizing their dimensions, weight, and texture (Table 6). The weight and thickness of the biscuits obtained did not differ significantly across the variants (α = 0.05); however, it was found that the addition of CP caused an increase in the diameter of the biscuits. Gluten proteins present in WF (replaced with CP) are responsible for the proper consistency and structure of the dough [70,71]. The observed increase in diameter may be caused by a reduction in the content of gluten proteins in the dough, which does not maintain its shape during preparation and baking. One of the parameters describing the quality of shortcake biscuits is the spread ratio. The larger the diameter to thickness ratio, the better the biscuit quality [72]. The overall spread ratio increased with the addition of CP and ranged from 6.30 for R to 7.75 for CP10. A significantly lower spread ratio in the case of R may result from a stronger binding by the action of gluten proteins, creating a dough with higher compactness. Literature data indicated that the addition of vegetable proteins, which bind water and other biopolymers, reduced the spread ratio and, on the other hand, increased the thickness of the biscuits [73,74,75]. According to Kulkarnia et al. [76], an increase in the biscuits spread ratio may indicate a poor connection of the protein and carbohydrate networks in the biscuits. These two components are important nutrients, but from a physical point of view, their interaction with one another can cause changes in the hardness of the biscuits. As expected, it was noted that replacing WF with CP resulted in a successive reduction in the hardness of the biscuits from 29.44 N for R to 24.50 N for CP10. The reduction in hardness can be explained by the uneven mixing process and the potential uneven distribution of the added ingredients, which may result in limiting the availability of water for proteins, which should be hydrated during the preparation of the dough. Too little water or additional dough ingredients such as fat and sugar prevent the proteins from being properly hydrated. The dough from which the biscuits are made is high in both sugar and fat and low in water, resulting in a dough with a sticky and consistent character and, consequently, increased hardness [77,78]. These results are in line with other studies by Ho and Abdul-Latif [74] and Chauhan et al. [79] who noted that replacing WF with other flours, and thus reducing the amount of gluten in the dough, also resulted in a reduction in the hardness of the biscuits.

### 2.5. Water Behavior

**Figure 3:**
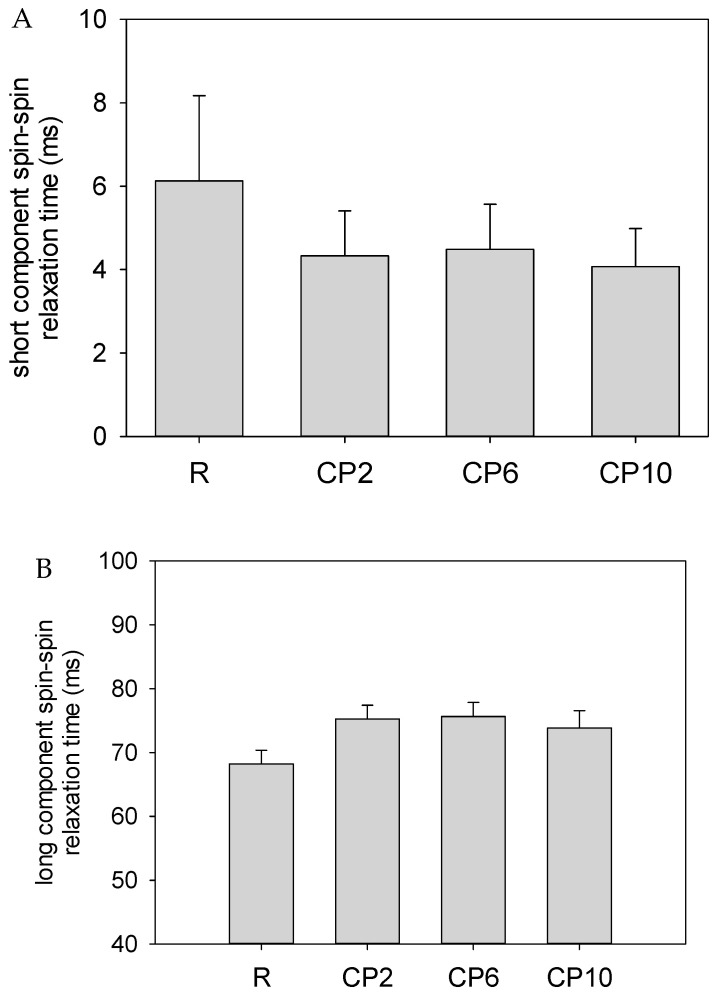

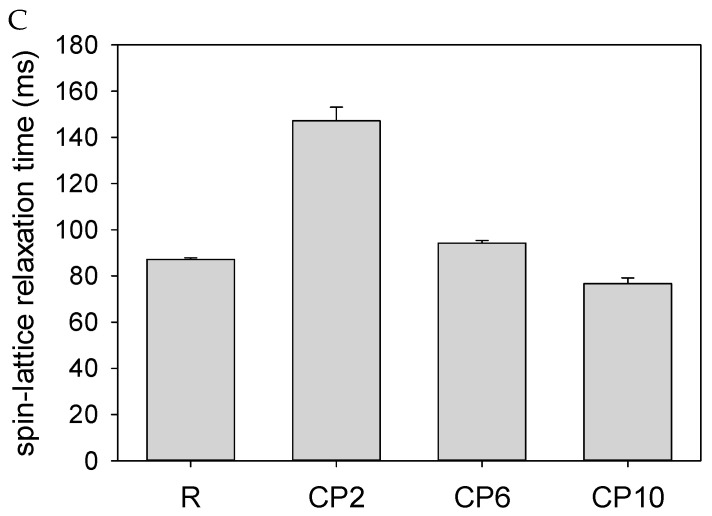
Results of relaxation times. R—reference biscuits; CP2, CP6, and CP10—biscuits with 2%, 6%, and 10% of wheat flour replacement by CP, respectively. (**A**)—results of short component spin–spin relaxation time. (**B**)—results of long component spin–spin relaxation time. (**C**)—results of spin–lattice relaxation time.

Measurements of the relaxation parameters revealed two CPMG (Carr-Purcell-Meiboom-Gill) proton populations and one FID (free induction decay) proton population. This is expected for low moisture products that are rich in carbohydrates and fats. In fresh dough samples with water content significantly above 50%, up to three CPMG proton populations can be observed T_21_ (<10 ms), T_22_ (20–50 ms) and T_23_ (>100 ms), namely tightly, less tightly, and weakly bound water, respectively. As the water content in dough decreases below 50%, the T_23_ component disappears, as there is no longer an excess of water in the system. Moreover, T_21_ and T_22_ components tend to merge, forming one proton population [38]. This is not the case for shortcake biscuits, as both short T_21_ (Figure 3A) and long T_22_ (Figure 3B) components of spin–spin relaxation time could be observed. Shortcake biscuits are characterized by a very low water content <2%, so one can expect that it will be bound very “tightly”, meaning that the T_21_ will correspond to the amount of water present in the system. Therefore, T_22_ will rather correspond to the amount of starch and fat in the system as those ingredients are present in large quantities and are the most proton abundant. This is in accordance with literature data, as for pure fat or fat in emulsion, relaxation times are estimated between 40–100 ms [36], whereas for pure starch the relaxation time may range between 40–180 ms (depending on water content) [80]. The presence of one spin–lattice relaxation time, T_1_ (Figure 3C), is once again conditioned by the low amount of water in the system. Starches at hydration levels below 10% are characterized by only one component of spin–lattice relaxation time; above that value, when bulk water starts to be present in the system, a long component of relaxation time T_12_ can be separated [80].

A reduction in the value of short components of the spin–spin relaxation times T_21_ is observed in the samples containing CP, compared to the reference sample R. This indicates limiting the dynamics of water molecules bound to the polymer matrix. This phenomenon may be attributed to the inclusion of cricket proteins as the behavior of water in food is significantly affected by the solubility of proteins, which consists of hydrophobic (protein–protein) and hydrophilic (protein–solvent) interactions [81]. Literature data indicate that CP is hydrophilic in nature [82], which limits the amount of water hydrating the proteins and starch of WF [22]. This corresponds to changes in firmness (Table 6), as it has followed the same manner as T_21_, suggesting that the addition of CP that causes decrease of water mobility results in softer texture of obtained biscuits, which were more fragile.

In contrast to short components, the long components of spin–spin relaxation time increased in samples where part of the WF was replaced with CP. This is the effect of an increase in fat content in samples containing more CP. The lack of a further increase in T_22_ with the increase in CP should be attributed to an overall lower amount of carbohydrates and fats. This is because of the fact that fat is a more proton-dense ingredient than starch, whereas a 10% replacement of WF with CP results in an over 5% reduction in sum of carbohydrates and fats.

From the point of view of the molecular properties of water, replacing a part of WF with CP reduces the binding of H_2_O molecules with biopolymers. This is normally manifested by an increase in the value of spin–lattice T_1_ relaxation times [83,84]; however, in shortcake biscuits, water molecules are present in relatively small quantities in comparison to starch or fat.

The smallest 2% replacement of WF with CP caused a significant increase in the T_1_ value compared with the R. This should be normally interpreted as an increase in the amount of bulk water compared to bound one, but CP2 was characterized by the lowest water content, so one should assume that any water present will be completely bound. In the samples CP6 and CP10, the values were comparable with those observed for R. This result does not allow for an unambiguous interpretation of the effect of CP and the removal of part of the WF on quantitative changes in water binding in the recipe-modified cookie; however, these irregular changes in the values of spin-lattice relaxation times are confirmed by the results of the equilibrium analysis of the water activity (a_r_) of the biscuits (Table 7). Taking into consideration the changes in water activity, water content, and spin–lattice relaxation time, it can be concluded that the sole implementation of CP in the recipe of WF shortcake biscuits causes interactions that decrease the binding of water at a molecular level. However, an increase in the replacement ratio of WF to CP reverses this effect. Although, due to low water content in the final product, this phenomenon was not reflected in texture analysis, it was noticed by consumers, as indicated by texture acceptance.

**Figure 4:**
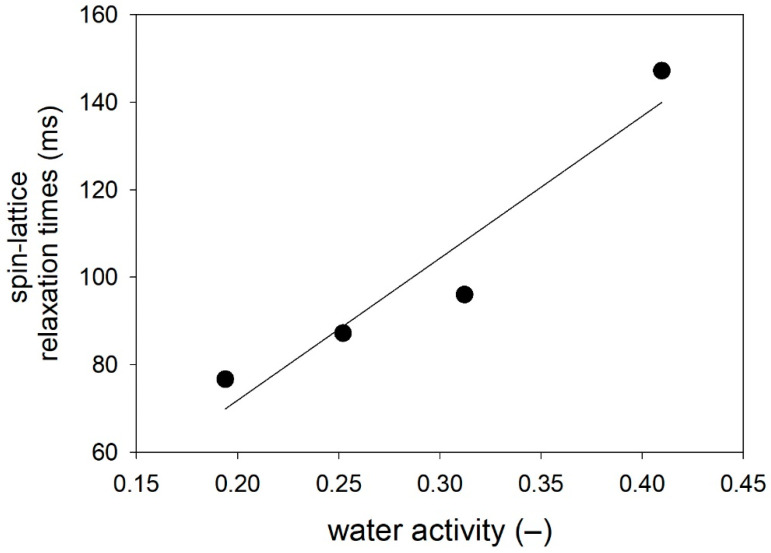
Correlation between water activity and spin–lattice relaxation times T_1_ (Pearson r = 0.900; *p* = 0.050).

**Figure 5:**
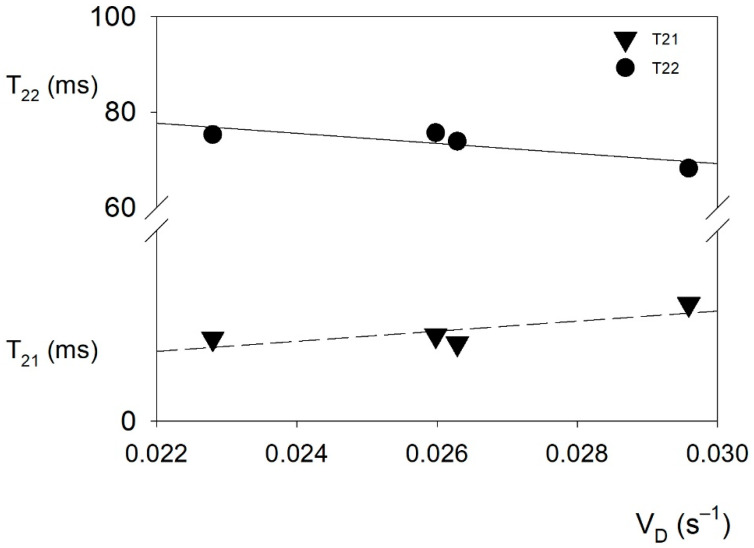
Linear correlation between transport rate (V_D_) and short (T_21_) and long components (T_22_) of the spin–spin relaxation times. (Pearson r = −0.849; *p* = 0.076 and r = 0.801; *p* = 0.100 for T_21_ and T_22_, respectively).

A correlation was found between T_1_ and a_r_ (Figure 4). The increase in the equilibrium water activity in the product determines the increase in the amount of bulk water compared to the amount of bound water. The mobility of the molecules of both water fractions is reflected in the values of the spin–spin relaxation time components. Linear correlations were found between the transport rate of water in the system (V_D_) and the mobility of rotational movements of bulk and bound water molecules (Figure 5). As the translational movement rate of the water molecules in the product increases, the possibility of rotational movements of the water molecules in the bulk fraction decreases, and at the same time, the bound fraction molecules achieve a greater possibility of rotational movements around the water–polymer matrix bond.

## 3. Materials and Methods

### 3.1. Shortcake Biscuits Manufacturing

The recipe for reference biscuits (denoted as R in the text) was as follows: 200 g wheat flour (type 500) (GoodMills Polska sp. z o.o., Grodzisk Wielkopolski, Poland), 64 g white sugar (Pfeifer & Langen Polska S.A., Środa Wielkopolska, Poland), 20 g brown sugar (Pfeifer & Langen Polska S.A., Środa Wielkopolska, Poland), 2 g milk powder (SM Mlekovita, Wysokie Mazowieckie, Poland), 2.5 g salt (Kopalnia Soli ‘Kłodawa’ S.A., Kłodawa, Poland), 2 g baking powder (Dr. Oetker Polska Sp. z o.o., Gdańsk, Poland), 80 g baking margarine (Upfield Polska sp. z o.o., Warsaw, Poland), and 44 g water. In the test samples, wheat flour was replaced with cricket powder (Crunchy Critters, Derby, UK) in three different quantities of 2%, 6%, and 10% (*w*/*w*) and denoted as CP2, CP6, and CP10, respectively. The amounts of other components were unchanged. The composition of CP (determined in a previously published study [16]) is: protein 42.0 ± 0.4 [%]; fat 29.1 ± 0.6 [%]; 3.6 ± 0.3 [%]; fiber 3.5 ± 0.02; and carbohydrate 21.8 ± 0.8 [%]. All the dry compounds were mixed together with the KitchenAid mixer (5KPM5EWH, KitchenAid, Greenville, OH, USA) for 3 min. Water was then added and mixing continued for another 1 min. The dough was rolled into 2 mm thick sheets, rounded shapes were cut with a Ø60 mm cookie cutter and placed in an aluminum tray. The position of biscuits on baking trays was the same for all variations. Biscuits were baked at 205 °C (MIWE Condo, MIWE Michael Wenz GmbH, Amstein, Germany) for 11 min, and then allowed to cool at room temperature for 15 min. The cool biscuits were packed in polypropylene pouches and stored at room temperature in darkness until analysis.

### 3.2. Consumer Acceptance

The rating of consumer acceptance was assessed by using the 9-point hedonic line scale (ranging from 1 “dislike very much” to 9 “like very much”) [85]. In this study, sixty-five untrained panelists, aged between twenty-six and forty-five, were invited to participate. The study involved 29 men and 36 women, students and employees of the Poznań University of Life Sciences (Poznań, Poland), who expressed a voluntary willingness to participate. Consumers were asked to evaluate the appearance, flavor, taste, texture, and overall rating of analyzed biscuits.

### 3.3. Color Measurements

A Chroma Meter CR-410 (Konica Minolta Sensing Inc., Tokyo, Japan) was used for the color measurements of biscuits [20]. Differences in color were recorded in CIE L*a*b* scale in terms of lightness (L*) and color (a*—redness; b*—yellowness). Analysis was repeated 10 times for each sample. The total color difference (ΔE) and whiteness index (WI) was calculated, with the R values used as baseline for all CP variants:

$$\begin{array}{c} \Delta E=\sqrt{{\Delta L*}^{2}+{\Delta a*}^{2}+{\Delta b*}^{2}}\\ \mathrm{WI}=100-\sqrt{{\left(100-L*\right)}^{2}+{a*}^{2}+{b*}^{2}} \end{array}$$

### 3.4. Proximate Composition and Energy Value

Determination of the moisture was carried out in accordance with the AACC 44-19.01 method [86]. Total nitrogen content was determined by the Kjeldahl method according to ISO 20483 [87] and was used to calculate the protein content by multiplying the result by the conversion factor of 5.7, suitable for wheat [88] and recommended by Ritvanen et al. [89] for crickets. The fat content was determined (Soxhlet method) according to AACC 30-25.01 [90], and ash content according to AACC Method 08-12.01 [91]. Moreover, the proximate carbohydrate content was estimated by subtracting the total fat, protein, ash, and moisture content from 100%. The ash, carbohydrate, fat, and protein contents were presented on a dry weight basis. The energy value (EV) was calculated with the following formula:

EV (kcal/100 g) = 4 × protein (%) + 4 × carbohydrate (%) + 9 × fat (%)

### 3.5. Minerals Content

The concentrations of the minerals Ca, Cu, Fe, K, Mg, Mn, Na, and Zn were determined using flame atomic absorption spectroscopy (F-AAS; SpectrAA-800, Varian, Palo Alto, CA, USA) preceded by microwave mineralization with nitric acid [92]. The recommendations for Ca, Cu, Fe, Mg, Mn, and Zn were established at the level of Nutrient Reference Value (NRV) [93]. The contents of minerals were expressed in g/100 g of the sample.

### 3.6. Amino Acid Composition

Samples before analysis of the amino acid profile were subjected to acidic hydrolysis in 6 M HCl under nitrogen at 110 °C for 24 h with modifications as reported by Kwanyuen and Burton [94]. The contents of amino acids were determined as derivatives of phenylisothiocyanate (PITC) according to the procedure described by Polanowska et al. [95] Norleucine (500 nM) was added as internal standard. The tryptophan content was examined after alkaline hydrolysis of proteins in 4 M NaOH at 110 °C for 18 h under nitrogen according to the method proposed by Çevikkalp et al. [96] The analysis was performed using LC Agilent Technologies 1200 Rapid Resolution (Santa Clara, CA, USA) system equipped with a UV-Vis detector DAD 1260 (Agilent Technologies, Santa Clara, CA, USA) and a reversed-phase column Zorbax Eclipse Plus C18 (4.6 × 150 mm, 5 µm) (Agilent Technologies, Santa Clara, CA, USA).

### 3.7. Fatty Acid Composition Analysis

Fat was extracted from the biscuits using the standard procedure described by Folch et al. [97] and the fatty acid composition was determined according to the AOCS Official Method Ce 1 h-05 [98] according to the parameters described in detail previously [54] with an Agilent 7820A GC (Agilent Technologies, Santa Clara, CA, USA) equipped with a flame ionization detector (FID) and SLB-IL111 capillary column (Supelco, Bellefonte, PA, USA) (100 m, 0.25 mm, 0.20 μm). The results were expressed as a percentage of total fatty acids.

### 3.8. Texture Analysis

A TA.XTplus texture analyzer (Stable Micro Systems Co. Ltd., Godalming, UK) equipped with a load cell of 5 kg was used to determine the texture properties of biscuits. Hardness was determined by the bend test, using the 3-point bend rig HDP/3PB (Stable Micro Systems Co. Ltd., Godalming, UK) with the following settings: pre-test speed of 5 mm/s, test speed of 3 mm/s, post-test speed of 10 mm/s, distance of 5 mm and distance between the supports of 2 cm [99]. At least 10 biscuit measurements were taken for each batch. Each biscuit was compressed once and maximum force was recorded.

### 3.9. LF NMR Relaxometry

Biscuit samples were placed in Ø8 mm measuring tubes. The height of the sample in the tube was set at 15 mm. After placing the samples, the tubes were closed with Parafilm^®^ and measurements were made.

^1^H NMR relaxation times (spin–lattice (T_1_) and spin–spin (T_2_)) were analyzed with a pulse NMR spectrometer PS15T operating at 15 MHz (Ellab, Poznań, Poland) at 21.0 ± 0.5 °C. The inversion–recovery (π − τ − π/2) [100] pulse sequence was applied for measurements of the T_1_ relaxation times. Distances between RF pulses (τ) were changed within the range from 0.5 to 50 ms and the repetition time was from 15 s. Each time, 32 FID signals and 110 points from each FID signal were collected. Calculations of the spin–lattice relaxation time values were performed with the assistance of the CracSpin program [101].

Measurements of the spin–spin (T2) relaxation times were taken using the pulse train of the Carr–Purcell–Meiboom–Gill spin echoes (π/2 − TE/2 − (π)_n_ [100]. The distance (TE) between π RF pulses ranged from 0.1 to 1.0 ms. The repetition time was 15 s. The number of spin echoes (n) amounted to 100. Five accumulation signals were employed. The calculations were performed by using the dedicated software by application of a non-linear least-square algorithm.

### 3.10. Water Activity

Rollers 1 cm thick and 2 cm in diameter, each cut from the tested product, were used for the measurements. The sample was placed in the measurement chamber. The analysis was performed by using water diffusion and activity analyzer ADA-7 (COBRABID, Poznań, Poland) with a sample temperature control panel. The analyzer is equipped with dedicated software to record temporary water activity during water evacuation process [34]. All measurements were performed at 21.0 ± 0.2 °C. The duration of one measurement was set to 1000 s. Based on the obtained curves, the equilibrium value of water activity a_w_ in the product and the transport rate V_D_ were determined. All presented results are mean values (*n* = 7) and standard deviation.

### 3.11. Statistical Analysis

Each biscuit variant was analyzed in three samples, with triple measurement of each, unless stated otherwise. One-way analysis of variance (ANOVA) was carried out independently for each dependent variable. A post hoc Tukey HSD multiple comparison test was used to identify statistically homogeneous subsets at α = 0.05. Moreover, the Pearson correlation coefficient was calculated between relaxation times and water activity parameters. Statistical analysis was performed with Statistica 13 software (Dell Software Inc., Round Rock, TX, USA).

## 4. Conclusions

Partial replacement of wheat flour with cricket powder in biscuits and other food products augmented their physical properties as well as their nutritional and functional values. A small (2%) addition of CP improved the ratings for flavor, texture, appearance, and the overall desirability of biscuits. However, further addition of CP (6% and 10%) resulted in significantly lower scores in consumer test. CP2 delivered 462 kcal, 7.8 g protein, 16.2 g fat, and 73 g carbohydrates in 100 g. Moreover, it had higher content of minerals: Ca (↑23%), Zn (↑16%), Mn (↑14%), Fe (↑12%), K (↑7%), and Mg (↑6%) than the commercial (control) product.

Changes were also observed in the physical properties of the biscuits. Replacing wheat flour with cricket powder resulted in a successive reduction in the hardness of cookies from 29.44 N for R to 24.50 N for CP10. A decrease in the values of the short components of the T_21_ spin–spin relaxation times was also observed in the samples containing CP compared to the reference sample R, measured by LF NMR, which indicates a reduction in the dynamics of water molecules bound to the polymer matrix. Due to the increase in the fat content of CP biscuits as opposed to the short ones, the long spin–spin relaxation time components increased in samples where some flour was replaced by CP. Nevertheless, on the basis of the results, it was found that the obtained shortbreads with a 2% CP addition could be successfully marketed. Moreover, the use of products with such superior characteristics as edible insects poses a viable scenario for the future demands of growing human population.

## Figures and Tables

**Table 1:** Color parameters of cricket powder and biscuits.

Parameter	CP	R	CP2	CP6	CP10
L*	52.04 ± 0.70	75.53 ± 0.28 ^a^	73.90 ± 0.28 ^b^	65.98 ± 0.29 ^c^	63.29 ± 0.16 ^d^
a*	6.02 ± 0.20	3.12 ± 0.05 ^d^	4.17 ± 0.08 ^c^	4.96 ± 0.02 ^b^	5.17 ± 0.06 ^a^
b*	14.65 ± 1.77	25.19 ± 0.93 ^a^	22.86 ± 0.37 ^b^	22.42 ± 0.53 ^b^	20.47 ± 0.12 ^c^
ΔE	-	-	3.03	10.11	13.28
WI	49.49	64.74	65.05	58.96	57.65

Mean values in biscuits samples with the same letters in the row (^a–d^) were not significantly different (α = 0.05). CP—cricket powder; R—reference biscuits; WI—whiteness index; CP2, CP6, and CP10—biscuits with 2%, 6%, and 10% of wheat flour replacement with CP, respectively.

**Table 2:** Proximate composition and energy value.

Parameter	R	CP2	CP6	CP10
Moisture (%)	1.75 ± 0.49 ^a^	1.65 ± 0.21 ^a^	2.08 ± 0.31 ^a^	1.75 ± 0.19 ^a^
Protein (%)	6.08 ± 0.08 ^d^	7.80 ± 0.24 ^d^	9.24 ± 0.18 ^b^	10.30 ± 0.09 ^a^
Fat (%)	14.7 ± 0.4 ^d^	16.2 ± 0.1 ^b^	16.8 ± 0.2 ^b^	17.5 ± 0.4 ^a^
Ash (%)	1.01 ± 0.12 ^b^	1.03 ± 0.03 ^b^	1.09 ± 0.15 ^b^	1.35 ± 0.09 ^a^
Carbohydrates ^1^ (%)	76.5 ± 1.14 ^a^	73.3 ± 1.03 ^c^	70.8 ± 1.01 ^b^	69.1 ± 1.15 ^d^
Energy value ^2^ (kcal/100 g)	454.5 ^d^	462.4 ^b^	461.5 ^b^	466.8 ^a^

^1^ The carbohydrate content was estimated by subtracting the average content of ash, fat, and protein from 100%. ^2^ Energy value was calculated based on the average moisture, protein, fat, and carbohydrate content. Mean values with the same letters in the row (^a–d^) were not significantly different (α = 0.05). R—reference biscuits; CP2, CP6, and CP10—biscuits with 2%, 6%, and 10% of wheat flour replacement with CP, respectively.

**Table 3:** Mineral composition (expressed as mg per 100 g of biscuits).

Mineral	NRV/AI (mg/Day)	R (mg/100)	CP2 (mg/100 g)	CP6 (mg/100 g)	CP10 (mg/100 g)
Ca	800	31.4 ± 1.8 ^d^	38.5 ± 2.0 ^c^	53.2 ± 0.9 ^b^	67.0 ± 3.2 ^a^
Mg	375	10.4 ± 0.1 ^d^	11.1 ± 0.2 ^c^	13.6 ± 0.1 ^b^	17.6 ± 1.0 ^a^
K	3500	102.6 ± 1.0 ^d^	109.8 ± 2.2 ^c^	137.8 ± 4.4 ^b^	152.3 ± 11.0 ^a^
Na	1500	323.1 ± 10.3 ^a^	310.2 ± 9.1 ^b^	302.0 ± 8.3 ^b^	310.6 ± 11.9 ^b^
Cu	1	0.021 ± 0.001 ^d^	0.044 ± 0.004 ^c^	0.106 ± 0.007 ^b^	0.196 ± 0.006 ^a^
Fe	14	0.536 ± 0.017 ^d^	0.602 ± 0.014 ^c^	0.662 ± 0.027 ^b^	0.786 ± 0.039 ^a^
Mn	2	0.191 ± 0.004 ^d^	0.216 ± 0.009 ^c^	0.310 ± 0.010 ^b^	0.365 ± 0.008 ^a^
Zn	10	0.706 ± 0.003 ^d^	0.819 ± 0.051 ^c^	1.23 ± 0.08 ^b^	1.61 ± 0.07 ^a^

NRV—nutrient reference value (for Ca, Mg, Cu, Fe, Mn, Zn), AI—adequate intake (for K, Na); Mean values with the same letters in the row (^a–d^) were not significantly different (α = 0.05). R—reference biscuits; CP2, CP6, and CP10—biscuits with 2%, 6%, and 10% of wheat flour replacement with CP, respectively.

**Table 4:** Fatty acid composition of biscuits enriched with CP (as a percentage of total fatty acids).

Fatty Acid	R	CP2	CP6	CP10
C 8:0	0.482 ± 0.002 ^a^	0.477 ± 0.016 ^b^	0.479 ± 0.013 ^b^	0.487 ± 0.001 ^a^
C 10:0	0.463 ± 0.005 ^b^	0.453 ± 0.013 ^b^	0.448 ± 0.009 ^a^	0.448 ± 0.005 ^a^
C 12:0	6.340 ± 0.002 ^a^	6.179 ± 0.064 ^b^	6.102 ± 0.085 ^b^	6.100 ± 0.015 ^b^
C 14:0	2.863 ± 0.004 ^a^	2.837 ± 0.008 ^b^	2.810 ± 0.013 ^b^	2.803 ± 0.002 ^b^
C 16:0	31.223 ± 0.019 ^b^	31.629 ± 0.063 ^a^	31.560 ± 0.033 ^a^	31.343 ± 0.197 ^b^
C 16:1	0.159 ± 0.045 ^a^	0.130 ± 0.001 ^b^	0.129 ± 0.003 ^b^	0.133 ± 0.001 ^b^
C 18:0	3.855 ± 0.029 ^c^	3.956 ± 0.004 ^b^	4.014 ± 0.027 ^b^	4.044 ± 0.001 ^a^
C 18:1	31.401 ± 0.024 ^a^	31.218 ± 0.025 ^b^	31.123 ± 0.080 ^b^	31.146 ± 0.120 ^b^
C 18:2	21.311 ± 0.032 ^a^	21.288 ± 0.061 ^a^	21.502 ± 0.049 ^b^	21.655 ± 0.042 ^c^
C 18:3	1.058 ± 0.021 ^c^	1.055 ± 0.046 ^c^	1.093 ± 0.006 ^b^	1.126 ± 0.008 ^a^
C 20:0	0.667 ± 0.010 ^a^	0.603 ± 0.005 ^b^	0.556 ± 0.007 ^c^	0.548 ± 0.065 ^c^
C 22:0	0.175 ± 0.016 ^b^	0.175 ± 0.025 ^b^	0.182 ± 0.030 ^a^	0.170 ± 0.002 ^b^
*Σ* SFA	46.069 ± 0.016	46.308 ± 0.131	46.151 ± 0.023	45.941 ± 0.152
*Σ* MUFA	31.561 ± 0.069	31.347 ± 0.024	31.253 ± 0.077	31.278 ± 0.118
*Σ* PUFA	22.370 ± 0.053	22.344 ± 0.107	22.596 ± 0.055	22.781 ± 0.034

Mean values with the same letters in the row (^a–c^) were not significantly different (α = 0.05). R—reference biscuits; CP2, CP6, and CP10—biscuits with 2%, 6%, and 10% of wheat flour replacement with CP, respectively.

**Table 5:** Amino acids profile expressed as mg per g of biscuits.

Amino Acid	R	CP2	CP6	CP10
*Essential amino acids*				
Histidine	1.48 ± 0.01 ^d^	1.64 ± 0.03 ^c^	1.90 ± 0.01 ^b^	2.05 ± 0.02 ^a^
Isoleucine	2.65 ± 0.17 ^c^	2.78 ± 0.20 ^c^	3.43 ± 0.08 ^b^	3.70 ± 0.06 ^a^
Leucine	4.91 ± 0.07 ^d^	5.44 ± 0.21 ^c^	6.37 ± 0.11 ^b^	6.65 ± 0.07 ^a^
Lysine	1.33 ± 0.05 ^d^	1.86 ± 0.03 ^c^	2.44 ± 0.05 ^b^	2.77 ± 0.01 ^a^
Cysteine	3.75 ± 0.08 ^d^	4.36 ± 0.01 ^a^	4.10 ± 0.05 ^b^	3.93 ± 0.02 ^c^
Methionine	1.12 ± 0.05 ^c^	1.32 ± 0.14 ^b^	1.51 ± 0.02 ^a^	1.57 ± 0.03 ^a^
Phenylalanine	3.31 ± 0.13 ^c^	3.79 ± 0.11 ^b^	4.04 ± 0.17 ^ab^	4.30 ± 0.11 ^a^
Tyrosine	2.27 ± 0.02 ^d^	2.65 ± 0.03 ^c^	3.12 ± 0.01 ^b^	3.39 ± 0.01 ^a^
Threonine	2.33 ± 0.02 ^d^	2.58 ± 0.07 ^c^	3.15 ± 0.01 ^b^	3.45 ± 0.02 ^a^
Tryptophan	0.102 ± 0.003 ^d^	0.138 ± 0.003 ^c^	0.356 ± 0.002 ^b^	0.399 ± 0.005 ^a^
Valine	2.93 ± 0.04 ^d^	3.18 ± 0.09 ^c^	4.07 ± 0.01 ^b^	4.48 ± 0.04 ^a^
*Σ* EAA *	26.18	29.74	34.49	36.69
*Dispensable amino acids*				
Alanine	1.23 ± 0.01 ^d^	1.78 ± 0.04 ^c^	2.30 ± 0.01 ^b^	2.94 ± 0.01 ^a^
Arginine	2.13 ± 0.06 ^d^	2.48 ± 0.05 ^c^	3.26 ± 0.02 ^b^	3.79 ± 0.02 ^a^
Aspartic acid	4.70 ± 0.11 ^d^	6.05 ± 0.04 ^c^	6.84 ± 0.05 ^b^	8.89 ± 0.06 ^a^
Glutamic acid	22.02 ± 0.12 ^d^	23.19 ± 0.28 ^c^	22.70 ± 0.08 ^b^	24.79 ± 0.08 ^a^
Glycine	2.14 ± 0.01 ^d^	2.53 ± 0.04 ^c^	3.16 ± 0.02 ^b^	3.64 ± 0.02 ^a^
Proline	6.86 ± 0.04 ^d^	7.23 ± 0.03 ^c^	7.87 ± 0.06 ^b^	8.08 ± 0.02 ^a^
Serine	3.76 ± 0.01 ^d^	4.41 ± 0.12 ^c^	4.73 ± 0.03 ^b^	5.12 ± 0.01 ^a^
*Σ* DAA *	42.84	47.67	50.86	57.25

* sums were calculated from the mean values. Mean values with the same letters in the row (^a–d^) were not significantly different (α = 0.05). R—reference biscuits; CP2, CP6, and CP10—biscuits with 2%, 6%, and 10% of wheat flour replacement with CP, respectively.

**Table 6:** Physical properties of biscuits.

Parameter	R	CP2	CP6	CP10
Weight (g)	7.21 ± 0.30 ^a^	7.55 ± 0.37 ^a^	7.54 ± 0.45 ^a^	7.46 ± 0.29 ^a^
Diameter (cm)	4.54 ± 0.21 ^b^	4.63 ± 0.30 ^b^	4.79 ± 0.17 ^ab^	4.88 ± 0.18 ^a^
Thickness (cm)	0.72 ± 0.06 ^a^	0.65 ± 0.05 ^a^	0.65 ± 0.07 ^a^	0.63 ± 0.06 ^a^
Spread Ratio (–)	6.30 ± 0.12	7.12 ± 0.03	7.40 ± 0.08	7.75 ± 0.09
Firmness (N)	29.44 ± 3.07 ^a^	25.44 ± 6.80 ^ab^	25.22 ± 5.16 ^ab^	24.50 ± 2.56 ^b^

Mean values with the same letters in the row (^a–b^) were not significantly different (α = 0.05). R—reference biscuits; CP2, CP6, and CP10—biscuits with 2%, 6%, and 10% of wheat flour replacement with CP, respectively.

**Table 7:** Results of water activity in biscuits.

Parameter	R	CP2	CP6	CP10
water activity a_r_ (-)	0.3123 ± 0.0012 ^b^	0.4098 ± 0.0012 ^a^	0.2522 ± 0.0038 ^c^	0.1940 ± 0.0008 ^d^
transport rate V_D_ (s^−1^)	0.0296 ± 0.0022 ^a^	0.0228 ± 0.0023 ^c^	0.0260 ± 0.0031 ^b^	0.0263 ± 0.0023 ^b^

Mean values with the same letters in the row (^a–d^) were not significantly different (α = 0.05). R—reference biscuits; CP2, CP6, and CP10—biscuits with 2%, 6%, and 10% of wheat flour replacement with CP, respectively.

## Data Availability

The datasets generated during and/or analyzed during the current study are available from the corresponding author on reasonable request.

## Sample Availability

Samples of cricket powder are commercially available on the market.
